# Gastrointestinal bleeding caused by splenic artery pseudoaneurysm in chronic pancreatitis: a case report and literature review

**DOI:** 10.1093/jscr/rjae574

**Published:** 2024-09-12

**Authors:** Chenyao Liu, Qingliang Zhu, Hailong Zhang, Zhongqiong Wang

**Affiliations:** Department of Gastroenterology, Southwest Medical University, Luzhou, Sichuan Province 646000, China; Department of Gastroenterology, The Affiliated Hospital of Southwest Medical University, Luzhou, Sichuan Province 646000, China; Department of Gastroenterology, The Affiliated Hospital of Southwest Medical University, Luzhou, Sichuan Province 646000, China; Department of Gastroenterology, The Affiliated Hospital of Southwest Medical University, Luzhou, Sichuan Province 646000, China

**Keywords:** chronic pancreatitis, splenic artery, pseudoaneurysm, gastrointestinal bleeding, angiography, endovascular embolization

## Abstract

Pseudoaneurysm of the splenic artery is a rare vascular complication of chronic pancreatitis, with a high mortality rate. Haemorrhage and abdominal pain are the most common manifestations, and so far there are no literature reviews on the rare complication of splenic artery pseudoaneurysm due to chronic pancreatitis. Therefore, we describe a male patient with worsening haemochezia and upper abdominal pain, who had been hospitalized repeatedly for ‘pancreatitis’ 1 year ago, and relevant investigations confirmed a pseudoaneurysm of the splenic artery, which was successfully treated by transarterial embolization, and a literature review is also presented.

## Introduction

Chronic pancreatitis (CP) is local or diffuse progressive inflammation of the pancreas due to various causes, accompanied by irreversible damage to the internal and external secretory functions of the pancreas. In the development and progression of CP, there may be false cyst formation, mechanical obstruction of the gastrointestinal tract and common bile duct, pancreatic ascites, pleural effusion, splenic vein thrombosis with portal hypertension and subsequent variceal bleeding, pseudoaneurysm formation and other complications. A true aneurysm involves all layers of the vessel wall, whereas a pseudoaneurysm is caused by a disruption of the arterial wall followed by a periarterial haematoma [1]. Pseudoaneurysm, mainly caused by digestive enzyme erosion of vessels near the pancreas, is a rare and life-threatening complication if pseudoaneurysm bleeding occurs. Once pancreatic pseudoaneurysms rupture, the mortality rate can exceed 40% [1]. Pseudoaneurysms are more often associated with CP compared to acute pancreatitis [2].

Blood vessels susceptible to pseudoaneurysm include splenic artery (35%–50%), gastroduodenal artery (20%–25%), pancreatic duodenal artery (20%–25%), and other arteries (5%) [3].

When pseudoaneurysm is suspected, early diagnosis and treatment are critical to prognosis. In CP complicated by splenic artery pseudoaneurysm (SAP), the most common symptoms are abdominal pain, bloody stool, or vomiting, but these symptoms are not specific. Therefore, the diagnosis is best established with gastroscopy, ultrasound, and computed tomography (CT). However, the most commonly used and most reliable study is angiography, which allows the benefit of transcatheter embolization in appropriate patients [4]. Related literature also indicates the temporary and precise effectiveness of arterial embolization in controlling bleeding in CP-associated pseudoaneurysms [5].

## Case report

A 66-year-old man presented to our hospital for worsening haematochezia and upper abdominal pain. He had a history of diabetes for 4 years, coronary artery disease for 2 years, and hypertension for 1 year. He was hospitalized repeatedly for ‘pancreatitis’ 1 year ago and had left pancreatic pseudocysts. On physical examination, his vital signs were stable, and there was mild tenderness under the xiphoid process and in the left abdomen.

**Figure 1:**
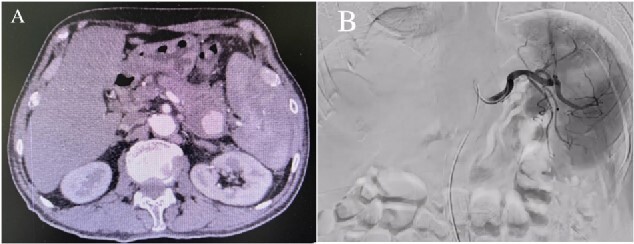
(A) Abdominal CT scan showed the presence of a SAP. (B) Celiac angiography confirmed that the main trunk of the splenic artery showed contrast agent overflow and the formation of a pseudoaneurysm of the splenic artery.

**Figure 2:**
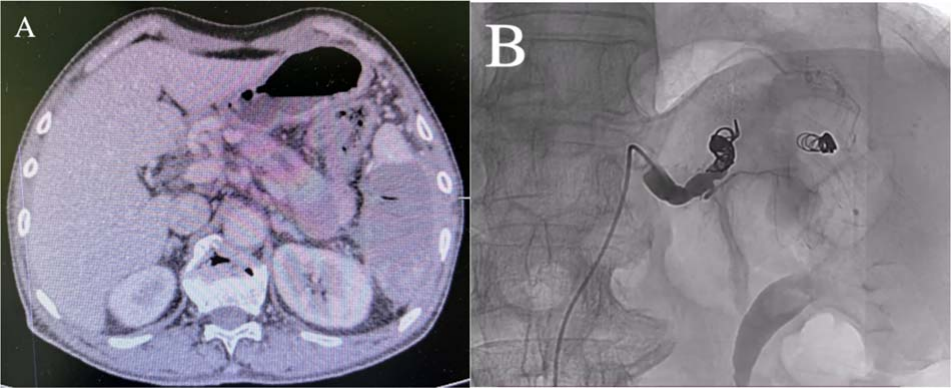
(A) Abdominal CT scan 6 days after the embolization showed no filling of the pseudoaneurysm. (B) After embolization, re-examination showed that the main trunk of the splenic artery was accurately occluded, and the staining of the pseudoaneurysm of the splenic artery disappeared.

Enhanced CT showed regional portal hypertension and CP invading the splenic artery and forming a pseudoaneurysm of the splenic artery, partially invading the jejunum (Fig. 1A). Subsequent celiac angiography confirmed a pseudoaneurysm originating from the splenic artery (Fig. 1B). Combined with the characteristics of the patient’s history and the results of angiography, the patient was diagnosed with gastrointestinal bleeding: pseudoaneurysm of the splenic artery and CP. After excluding relevant contraindications, abdominal arteriography and embolization were performed simultaneously. So further intubation until confirmation of splenic artery angiography, we used a coil to embolize the pseudoaneurysm. At that time, repeat angiography showed that the main splenic artery and the pseudoaneurysm showed no further enhancement (Fig. 2B). Subsequent CT scan 6 days after the embolization showed no filling of the pseudoaneurysm (Fig. 2A), and he was discharged 2 days later. After 6 months of follow-up, the patient recovered well and returned to normal work and life.

## Discussion

**Figure 3:**
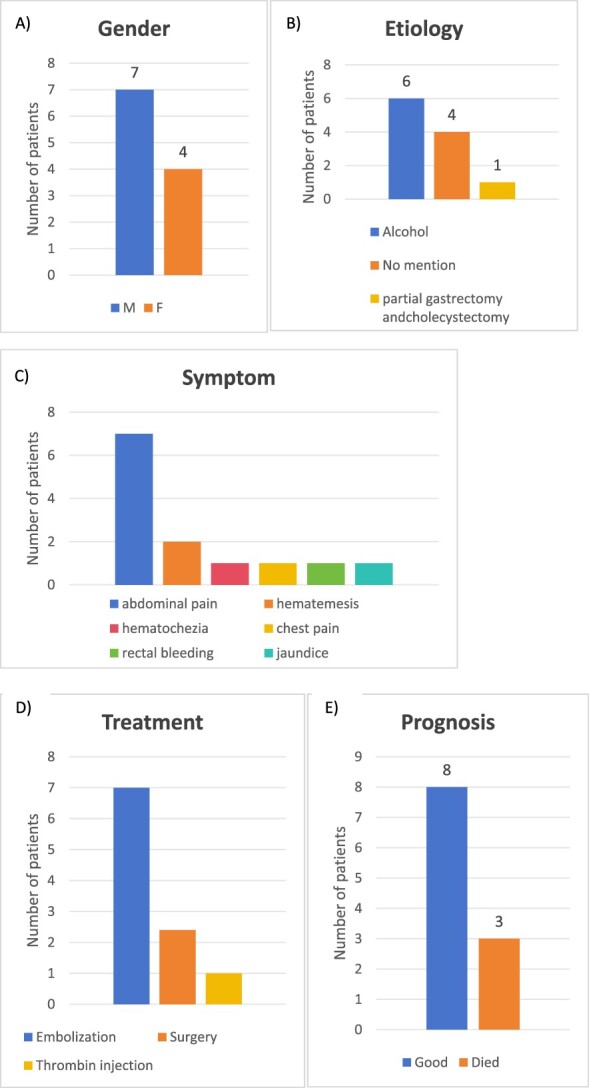
Graphical summary of the literature review. (A) Gender distribution, (B) aetiology of CP, (C) treatment, (D) aetiology of pancreatitis, and (E) outcome of 12-published cases (2014–2024) of SAP caused by CP.

We present a successful case of interventional treatment of gastrointestinal bleeding caused by a SAP in CP. Gastrointestinal bleeding in patients with pancreatitis has several causes, including bleeding from a pancreatic pseudocyst, a pseudoaneurysm, and portal or splenic vein thrombosis [1]. Unlike true aneurysms, the wall of a pseudoaneurysm does not contain arterial tissue. The release of lytic enzymes from the pancreas erodes the vessel wall, resulting in a periarterial haematoma. The risk of rupture is higher than that of a true aneurysm of comparable size due to the poor support of the aneurysm wall [6]. Although we searched PubMed from January 2014 to June 2024 using the keywords ‘pseudoaneurysm’ and ‘chronic pancreatitis,’ only 11 cases of SAP caused by CP were reported in the English literature [7–16] (Table 1). Their age/gender, symptoms, treatment, and prognosis are summarized in Table 2, and Fig. 3 provides a graphical summary of the published case reports.

**Table 1:** Summary of reported cases of SAP caused by CP.

Year	Author/Reference	Number of cases	Aetiology of CP	Imaging	Angiography
2024	Matthew Cunningham-Hill	1	Partial gastrectomy and cholecystectomy	Enhanced CT	Yes
2021	Hui Zhao	1	No mention	Enhanced CT	Yes
	Antonio Borzelli	1	Alcohol	Enhanced CT	Yes
2020	Jonathon N Holt	1	Alcohol	Enhanced CT	No
2020	Hytham K.S. Hamid	2	No mention	Enhanced CT	Yes No
2018	Paulo Roberto Prette	1	Alcohol	UDE	Yes
2018	Rickesh B Karsan	1	Alcohol	Enhanced CT	Yes
2018	Yumiko Yamanaka	1	No mention	SMI	Yes
2017	Mihai Adrian Eftimie	1	Alcohol	Enhanced CT	No
2016	Amit Shrivastava	1	Alcohol	Enhanced CT	Yes

CP, chronic pancreatitis; SAP, splenic artery pseudoaneurysm; CT, computed tomography; UDE, upper digestive endoscopy; SMI, superb microvascular imaging.

**Table 2:** Details of patients with SAP.

Year	Age/gender	Symptom	Treatment	Prognosis
2024	50/F	Upper abdominal pain	Embolization and thrombin injection	Good
2021	47/M	Sudden upper abdominal pain and jaundice	Embolization	Good
	45/M	Upper abdominal pain	Embolization	Good
2020	57/M	Acute left-sided abdominal pain	Surgery	Good
	58/F	Acute left-sided abdominal pain	Embolization	Good
	59/M	Sudden upper abdominal pain and rectal bleeding	Surgery	Died of sepsis
2018	48/F	Haematemesis	Embolization	Died of pulmonary sepsis
	40/M	Chest pain	Surgery	Good
	52/F	Hematemesis	Embolization	Good
2017	59/M	Haematochezia	Surgery	Died of MODS
2016	49/M	Upper abdominal pain and haematemesis	Percutaneous embolization	Good

SAP, splenic artery pseudoaneurysm; MODS, multiple organ dysfunction syndrome.

Review of the literature revealed that the most commonly involved vessels in pseudoaneurysm formation are the juxtapancreatic arteries, at first the splenic artery followed by the gastroduodenal artery and the pancreaticoduodenal arteries [1]. Digestive haemorrhage caused by pseudoaneurysm often manifests as intermittent bleeding and abdominal pain. The most common clinical symptoms are black stools, haematemesis, and haematochezia [17]. Alcoholic CP was the most significant predictor for the development of pseudoaneurysm.

It is difficult to differentiate pseudoaneurysm bleeding from other causes of digestive haemorrhage in patients with CP. Once a patient with pancreatitis experiences unexplained digestive haemorrhage, the diagnosis of a pseudoaneurysm should be considered and urgent further examination should be conducted. However, the early detection of a bleeding pseudoaneurysm is very significant for subsequent management. At this time, preprocedure imaging is integral to choosing appropriate endovascular treatment or surgery, such as ultrasound and enhanced CT and magnetic resonance imaging (MRI). Subsequent angiography is the gold standard for diagnosing pseudoaneurysm. Angiography can clearly identify the rupture position in an artery [18, 19].

The treatment of pseudoaneurysm complicated by gastrointestinal bleeding includes medical, interventional, and surgical management. However, due to advances in intravascular technology and hardware, endovascular embolization is the first-line treatment for arterial pseudoaneurysms in patients with pancreatitis [20].

When CP or pancreatic pseudocyst presents with gastrointestinal bleeding, regional portal hypertension is more common. However, the pseudoaneurysm caused by CP described in this case is a relatively rare cause of upper gastrointestinal bleeding. Therefore, through this case, whenever patients with pancreatitis or pseudocysts experience unexplained gastrointestinal bleeding, they should be alert to the possibility of pseudoaneurysm.

## Conclusion

SAP is a rare complication of CP, often associated with significant gastrointestinal bleeding. The possibility of pseudoaneurysm should be considered in patients with unexplained gastrointestinal bleeding. Its diagnosis relies mainly on imaging studies, and ultrasound, enhanced CT, or MRI. Angiography is valuable in localizing bleeding pseudoaneurysms, and endovascular embolization is the first-line treatment for arterial pseudoaneurysms in patients with pancreatitis.
